# Smartphone dependence classification using tensor factorization

**DOI:** 10.1371/journal.pone.0177629

**Published:** 2017-06-21

**Authors:** Jingyun Choi, Mi Jung Rho, Yejin Kim, In Hye Yook, Hwanjo Yu, Dai-Jin Kim, In Young Choi

**Affiliations:** 1 Department of Computer Science and Engineering, Pohang University of Science and Technology, Pohang, Republic of Korea; 2 Department of Medical Informatics, College of Medicine, The Catholic University of Korea, Seoul, Republic of Korea; 3 Department of Creative IT Engineering, Pohang University of Science and Technology, Pohang, Republic of Korea; 4 Department of Psychiatry, College of Medicine, Seoul St. Mary’s Hospital, The Catholic University of Korea, Seoul, Republic of Korea; Ariel University, ISRAEL

## Abstract

Excessive smartphone use causes personal and social problems. To address this issue, we sought to derive usage patterns that were directly correlated with smartphone dependence based on usage data. This study attempted to classify smartphone dependence using a data-driven prediction algorithm. We developed a mobile application to collect smartphone usage data. A total of 41,683 logs of 48 smartphone users were collected from March 8, 2015, to January 8, 2016. The participants were classified into the control group (SUC) or the addiction group (SUD) using the Korean Smartphone Addiction Proneness Scale for Adults (S-Scale) and a face-to-face offline interview by a psychiatrist and a clinical psychologist (SUC = 23 and SUD = 25). We derived usage patterns using tensor factorization and found the following six optimal usage patterns: 1) social networking services (SNS) during daytime, 2) web surfing, 3) SNS at night, 4) mobile shopping, 5) entertainment, and 6) gaming at night. The membership vectors of the six patterns obtained a significantly better prediction performance than the raw data. For all patterns, the usage times of the SUD were much longer than those of the SUC. From our findings, we concluded that usage patterns and membership vectors were effective tools to assess and predict smartphone dependence and could provide an intervention guideline to predict and treat smartphone dependence based on usage data.

## Introduction

Excessive smartphone dependence causes personal and social problems. There is considerable debate over smartphone dependence among adults and its consequent impact on health in a global context. Many researchers and nations are concerned about smartphone dependence. Various studies have been conducted to find factors associated with smartphone dependence and to prevent and manage smartphone dependence [1–6].

In particular, the provision of an appropriate intervention is important for the prevention and management of smartphone dependence. To provide an appropriate intervention for smartphone dependence, the degree of risk needs to be assessed using diverse data, including surveys, diaries, medical records, usage data, and positional or physiological monitoring data. Currently available screening and intervention data are based on self-reported surveys that may contain confounding information. Thus, the following issues must be considered for these analyses: what data to collect, how to collect them, and how to use them.

Recent information technology developments have opened the door to new approaches, including remote usage monitoring data and data mining tools. Smartphones also enable the easy collection of monitoring data and thus serve as an ancillary monitoring device. Therefore, smartphone usage data are valuable and easy to collect, and assessments based on real-world data are important for personalized prevention and management.

Additionally, a large-scale real-world data analysis requires a machine learning technique due to the size of the data. Large-scale data must be decomposed into meaningful concepts (i.e., usage patterns or phenotypes) to reduce the problem and obtain effective predictions. Tensor factorization is a well-known dimensionality reduction method that is used to derive meaningful concepts from large-scale electronic health record (EHR) data [7–9]. Tensor factorization is a powerful method because it can capture relationships in high-dimensional data [8].

Accordingly, the aim of this study was to derive usage patterns that were directly correlated with smartphone dependence from usage data, including Apps and timeslots. Additionally, we attempted to predict smartphone dependence using a data-driven prediction algorithm.

## Materials and methods

Our data analysis procedure consisted of the following three steps: 1) collection of smartphone usage log data, 2) derivation of smartphone usage patterns via tensor factorization, and 3) prediction of smartphone dependence based on the patterns.

### 2.1 Collecting smartphone usage log data

First, we developed a smartphone usage monitoring application [10] to collect smartphone usage log data. Smartphone users could freely download the smartphone usage monitoring application, which only supported Android, from Android application stores. Once the application is installed and executed, it runs as a background application to monitor usage. If smartphone users permit access to application usage by tapping “on” on the application access screen, data are sent to the MindsCare PC application [10]. After the data are successfully stored and integrated, we can monitor smartphone usage log data in the MindsCare PC application. The development languages and tools used to develop the smartphone usage monitoring application were Android native, Android SDK, JavaScript Object Notation (JSON), and Google's Android Open API. Smartphone usage log data were collected with the application event collection API (UsageEvents, AppOpsManager, and UsageStatsManager), which were supported after Android OS 5.0 (Lollipop). Details on the smartphone usage monitoring application can be found in a previous work [10].

For this study, 62 participants were recruited by a professional polling company (Hankook Research, Inc.) from November 26 to December 26, 2014. However, 10 participants were excluded because their smartphones did not support Android applications. Additionally, four participants were excluded from our data analysis because they uninstalled or forced our monitoring application to stop, which resulted in unsuccessful log collection.

**Fig 1:**
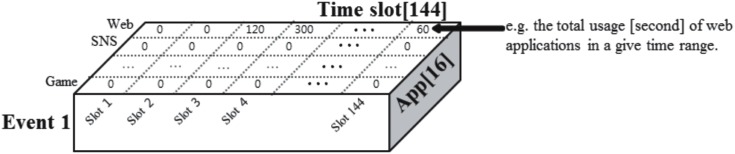
Example event of a smartphone user.

The log data were collected from 48 smartphone users through our developed application. Each user was classified into either the control group (SUC) or the addiction group (SUD) using the Korean Smartphone Addiction Proneness Scale for Adults (S-Scale) [11] and a face-to-face offline interview with a psychiatrist and a clinical psychologist [12]. The S-Scale has the advantage of reflecting Korean society [13]; its 15 questionnaire items are listed in S1 Appendix [11]. The Korean version of the Mini International Neuropsychiatric Interview (MINI) was used for the structured interview [14]. We preprocessed the collected log data to obtain events. One event contained information on how much the user used particular Apps every 10 minutes over the course of one day. Fig 1 shows one event for a specific user.

A user could have multiple events, because the log data were collected over several days from March 8, 2015, to January 8, 2016. Finally, we collected 760 events for 16 Apps × 144 time slots. We aimed to identify the time slots and Apps that were used primarily during the day. For the remainder of this study, we analyzed the events by representing the users as events.

### 2.2 Deriving smartphone usage patterns via tensor factorization

**Fig 2:**
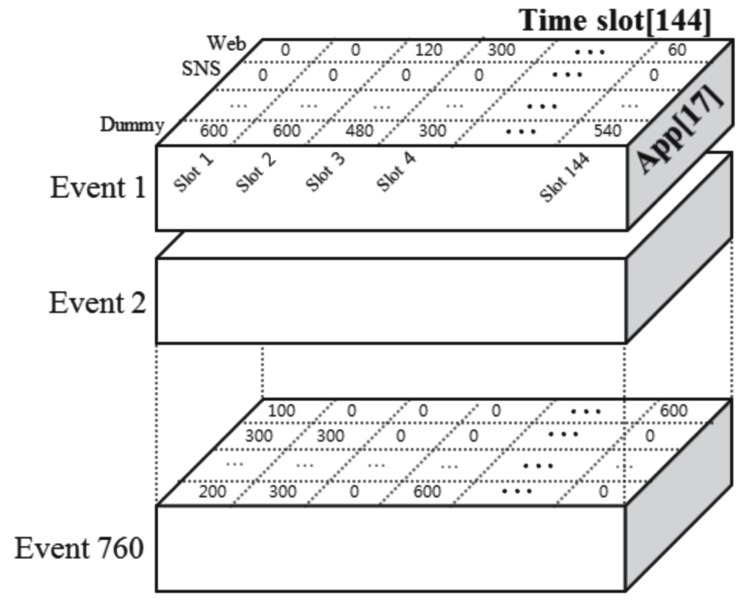
Constructed tensor from the events.

First, we introduced a *dummy* App to indicate the unused time for each time slot. The usage time of the dummy App was defined as 10 minutes–the sum of the usage time from 16 Apps. Therefore, we transformed the collected events into the events for 17 Apps (including the dummy category) × 144 time slots. Then, we represented the two-dimensional events in a three-dimensional tensor. A tensor is a natural representation of high-dimensional data [15]. We constructed a three-dimensional tensor for multiple events to analyze the interaction between the time slots and the Apps among the events (Fig 2).

**Fig 3:**
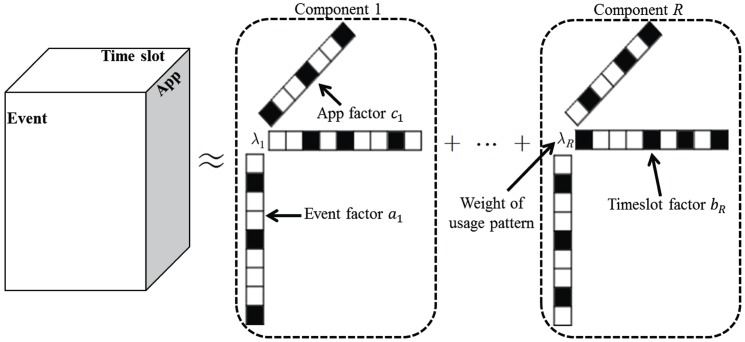
Generating usage patterns via tensor factorization. Each component consists of the event factor *a*_*r*_, the time slot factor *b*_*r*_, the App factor *c*_*r*_ and the weight *λ*_*r*_.

To derive usage patterns, we decomposed the constructed tensor *X* into *R* components using CP-APR tensor decomposition [16] as follows: $X\approx \sum_{r=1}^{R}\lambda_{r}a_{r}{}^{\circ}b_{r}{}^{\circ}c_{r}$, where *a*_*r*_, *b*_*r*_, and *c*_*r*_ are vectors, *λ*_*r*_ is a scalar, and ° is the outer product of the vectors (Fig 3). More details about tensor factorization can be found in S2 Appendix.

Using *b*_*r*_ and *c*_*r*_, we defined the *r*th usage pattern that represented the time slots and Apps that were mainly used during the day. *b*_*r*_ represents how much the 144 time slots are involved in the *r*th pattern. Similarly, *c*_*r*_ represents how much the 17 Apps are involved in the *r*th pattern. *λ*_*r*_*a*_*r*_ contains the degree to which the events have usage time associated with the *r*th pattern. *λ*_*r*_*a*_*r*_ was defined as the membership values of events to the *r*th pattern. Using the patterns, one event (17 Apps × 144 time slots) can be expressed as *R* membership values. We defined the set of *R* membership values for an event as a *membership vector*. After deriving the patterns, we used forward feature selection [17] to delete patterns that were not significant for the prediction of smartphone dependence. Details on the vector representation of an event via CP-APR can be found in previous reports [7,8,16]. We used the *cp_apr* function in the Matlab Tensor Toolbox [18].

### 2.3 Predicting smartphone dependence according to patterns

We predicted whether an event was from an addicted user using logistic regression. The membership vectors were used as the features of the prediction model. We used accuracy, recall, precision, and the area under the curve (AUC) of the prediction model to evaluate the prediction performance. Accuracy is defined as the ratio of true predictions, and recall is considered the ratio of the events that are correctly predicted among the actually addicted cases. Precision is defined as the ratio of the events from actually addicted users among the predicted events from addicted users. We performed the prediction with stratified 10-fold cross validation.

### 2.4 Ethics

This study was approved by the Catholic University’s Institutional Review Board (IRB number: KC15EISI0103). The data from the participants were de-identified. All users provided written informed consent prior to taking part in the study.

## Results

### 3.1 Collecting smartphone usage log data

The participants included 48 smartphone users from Seoul and Gyeonggi-do Province in South Korea (Table 1). Approximately 52.1% of the participants were classified in the addiction group. The participants’ ages ranged from the 20 to 39 years. A total of 25 participants were in their 20s (control group = 11 and addiction group = 14), whereas 23 participants were in their 30s (control group = 12 and addiction group = 11). There were 29 males (control group = 17 and addiction group = 12) and 19 females (control group = 6 and addiction group = 13).

**Table 1:** Demographic characteristics of the participants.

Variable	Category	SUD N (%)	SUC N (%)
Residence	Seoul	12 (48.00)	12 (52.17)
	Gyeonggi-do Province	13 (52.00)	11 (47.83)
Gender	Male	12 (48.00)	17 (73.91)
	Female	13 (52.00)	6 (26.09)
Age	20–29	14 (56.00)	11 (47.83)
	30–39	11 (44.00)	12 (52.17)
Educational level	College graduate or lower	12 (48.00)	5 (21.74)
	College graduate or higher	13 (52.00)	18 (78.26)
Marital status	Single	17 (68.00)	15 (65.22)
	Married	8 (32.00)	8 (34.78)
Occupational status	Office job	7 (28.00)	9 (39.13)
	Student	9 (36.00)	4 (17.39)
	Other	9 (36.00)	10 (43.48)
Total		25 (100.00)	23 (100.00)

Note: *N* = the number of participants

For each participant, log data were collected for an average of 15.8 days. In total, we had 760 events with 41,683 non-zero values for 760 events × 144 time slots × 16 Apps. Among the 760 events, 381 events (50.1%) were from the addiction group and 379 events (49.9%) were from the control group. We observed 876 Android programs and grouped them into 16 Apps according to their properties (Table 2).

**Table 2:** Observed Android programs.

**Apps**	**SNS**	**Health/** **Exercise**	**Game**	**Education**	**Trans-** **portation**	**Finance**	**Weather**	**Decoration**
**# program**	32	12	92	10	24	79	5	19
**Apps**	**Tool/** **Productivity**	**Lifestyle**	**Business**	**Pictures**	**Shopping**	**System**	**Enter-** **tainment**	**Web**
**# program**	106	62	39	22	45	218	100	11

Note: # program = the number of Android programs

### 3.2 Deriving smartphone usage patterns via tensor factorization

**Fig 4:**
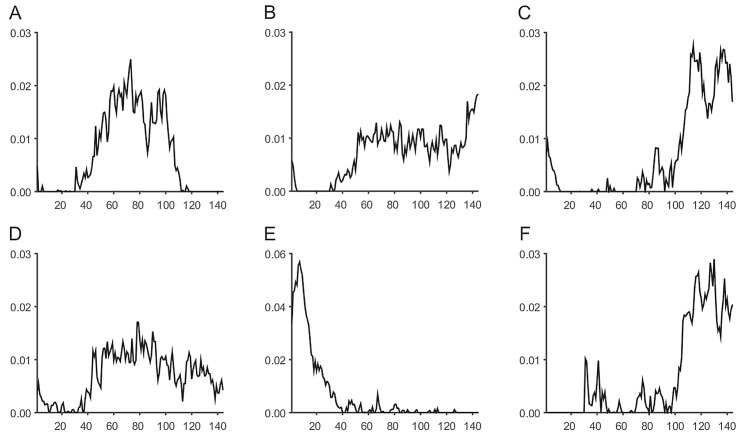
Visualization of the six patterns. (A) SNS during daytime, (B) web surfing, (C) SNS at night, (D) mobile shopping, (E) entertainment, and (F) gaming at night. The x-axis is the time slot (10 minutes), whereas the y-axis represents the involvement in time slots.

We derived six patterns after tensor factorization and forward feature selection by setting *R* = 10. These six patterns were as follows: social networking services (SNS) during daytime, web surfing, SNS at night, mobile shopping, entertainment, and gaming at night (Table 3 and Fig 4). For each pattern, we visualized the involvement of the time slots and Apps to observe the time slots and Apps that were used primarily during the day. We reported the two most involved Apps for each pattern.

**Table 3:** Characteristics of the six patterns.

Pattern	Characteristics	
	Two most involved Apps	Ratio
**SNS during daytime**	SNS: Inv. = 0.55, Lifestyle: Inv. = 0.13	Events: 64.61% (491) Addiction group: 36.84% (280) Control group: 27.76% (211)
**web surfing**	Web: Inv. = 0.67, Shopping: Inv. = 0.20	Events: 61.32% (466) Addiction group: 35.92% (273) Control group: 25.39% (193)
**SNS at night**	SNS: Inv. = 0.65, Decoration: Inv. = 0.08	Events: 56.97% (433) Addiction group: 34.61% (263) Control group: 22.37% (170)
**mobile shopping**	Finance: Inv. = 0.83 Transportation: Inv. = 0.11	Events: 51.18% (389) Addiction group: 33.95% (258) Control group: 17.24% (131)
**entertainment**	Entertainment: Inv. = 0.26 SNS: Inv. = 0.24	Events: 33.16% (252) Addiction group: 19.87% (151) Control group: 13.29% (101)
**gaming at night**	Game: Inv. = 0.93 Tool/Productivity: Inv. = 0.03	Events: 25.79% (196) Addiction group: 16.45% (125) Control group: 9.34% (71)

*Note*: Inv. = Involvement in App

Additionally, we calculated the ratio indicating how many events were relevant to the pattern by counting the number of events with a membership value to the pattern that was larger than one and dividing by the total number of events (i.e., 760). The ratio for the addiction group was calculated using the same process. The six patterns were statistically significant (i.e., p-values < 0.05) for the prediction of smartphone dependence (Table 4). Positive coefficient estimates indicated that increasing the corresponding values of the membership vector raised the probability of smartphone dependence.

**Table 4:** Significance of the six patterns.

Patterns	Coefficients	Standard errors	*p*-value
**SNS during daytime**	7.25 × 10^−5^	1.95 × 10^−5^	0.0002^**^
**web surfing**	6.86 × 10^−5^	1.61 × 10^−5^	0.0000^**^
**SNS at night**	6.58 × 10^−5^	1.93 × 10^−5^	0.0006^**^
**mobile shopping**	-5.22 × 10^−5^	2.05 × 10^−5^	0.0109^**^
**entertainment**	5.77 × 10^−5^	1.77 × 10^−5^	0.0012^**^
**gaming at night**	4.35 × 10^−5^	1.64 × 10^−5^	0.0081^**^

Note

** significant (*p* < 0.05) at a 95% confidence level.

To observe differences in usage times between the SUD and the SUC, we identified the median value for the usage times of each pattern (Table 5). We excluded events that showed a membership value of the pattern of zero when finding the median value. For all patterns, the median value was much longer for the SUD than for the SUC. A significant difference between the two groups was found in the entertainment pattern. Entertainment Apps were mainly used at dawn (i.e., 12 AM—5 AM). The median value of the SUD showed a membership value for the entertainment pattern that was longer than the median value of the SUC between 12 AM and 5 AM. For the SUD and SUC groups, the median values of the entertainment patterns were 36.1 minutes and 10.5 minutes, respectively. In the mobile shopping pattern, we found no significant differences between the two groups. We also performed a t-test to compare the means of the usage times between the two groups. For all patterns, the means of both groups were not the same according to the t-values.

**Table 5:** Median values and t-test results for usage times of each pattern.

Patterns	SUD (minutes)	SUC (minutes)	Difference (minutes)	t-value (p)
**SNS during daytime**	29.3	8.9	20.4	8.136 (0.000^**^)
**web surfing**	21.7	8.1	13.6	3.968 (0.000^**^)
**SNS at night**	24.2	12.7	11.5	4.759 (0.000^**^)
**mobile shopping**	6.3	3.4	2.9	-3.781 (0.000^**^)
**entertainment**	36.1	10.5	25.6	5.421 (0.000^**^)
**gaming at night**	30.8	6.3	24.5	5.087 (0.000^**^)

Note

**p < 0.005

### 3.3 Predicting smartphone dependence according to usage patterns

Next, we demonstrated the effectiveness of the six patterns and membership vectors for the prediction of smartphone dependence. We compared our prediction results with the raw feature matrix with 760 × 2448 columns, in which each row consisted of an event (a 17 Apps × 144 timeslots matrix was converted into a 1 × 2,448 vector). Table 6 provides the means and standard deviations of the prediction performance over 10-fold cross validation.

**Table 6:** Prediction performance.

Data	Accuracy (%)	Recall (%)	Precision (%)	AUC
Raw data	69.341±5.951	61.97±6.346	74.985±11.787	0.70684±0.08
Membership vectors	**75.921**±**4.388**	**81.884**±**12.504**	**74.671**±**8.407**	**0.81831**±**0.046**

As a result, we found that the membership vectors of our patterns obtained a significantly better prediction performance than the raw data. The AUC obtained by the membership vectors was 0.8183, whereas the AUC of the raw data was only 0.7068. In terms of accuracy, the membership vectors achieved an increase of almost 6.5%. These results demonstrate the effectiveness of using the patterns and membership vectors.

## Discussion

This study derived usage patterns that were directly correlated with smartphone dependence using smartphone usage data, including Apps and timeslots. We classified smartphone dependence using a data-driven prediction algorithm. Based on the results of this study, we obtained the following conclusions.

First, we identified the six patterns of smartphone use as follows: 1) SNS during daytime, 2) web surfing, 3) SNS at night, 4) mobile shopping, 5) entertainment, and 6) gaming at night.

The SNS patterns were the dominant patterns, including SNS during daytime and SNS at night. According to the Seven Shades of Mobile report of the seven primary motivations for mobile usage, 19% of individuals show a primary motivation to socialize, such as interacting with other people [19]. In our study, we observed a difference in usage timeslots, with the usage time much longer for the SUD than for the SUC.

The second smartphone usage pattern was the web surfing pattern. In this case, users used the smartphone for constant web surfing all day. The usage time of the SUD was also much longer than the usage time of the SUC.

The third pattern was the mobile shopping pattern. The usage times of the mobile shopping pattern did not significantly differ between the two groups. In this study, mobile shopping Apps were mainly services related to purchasing goods, such as books, tickets, used cars, used market items, gifts, or game items, and accessing discount information. Smartphone users usually used an App for these functions. Shopping, such as looking for and purchasing a product, is an ordinary lifestyle [19].

The next pattern was the entertainment pattern. The users intensively used smartphones for entertainment from midnight to dawn (i.e., 12 AM - 5AM). The usage time of the SUD was also much longer than the usage time of the SUC between 12 AM and 5 AM. For the SUD and SUC groups, the median values of the entertainment patterns were 36.1 minutes and 10.5 minutes, respectively. The contents of the entertainment Apps included news, sports, books, cartoons, travel, music, video, and information sharing. This usage timeslot was unique from midnight to dawn, which highlighted an abnormal but important result.

Finally, we obtained interesting results for gaming at night. A significant difference between the two groups was found for time spent gaming at night. Game Apps were mainly used in the afternoon (i.e., 5 PM—12 AM). The SUD, which often had a membership value for gaming at night, used the game Apps longer than the SUC between 5 PM and 12 AM. In the SUD and SUC groups, the median values of the gaming at night patterns were 30.8 minutes and 6.3 minutes, respectively. Thus, the six usage patterns were identified as useful features for the classification of smartphone dependence.

Additionally, the membership vectors of the usage patterns showed a significantly better prediction performance than the raw data. The positive coefficient estimates (patterns of SNS during daytime, web surfing, SNS at night, entertainment, and gaming at night) indicated that higher corresponding values of the membership vector were associated with a greater probability of smartphone addiction and that there was a low probability of smartphone addiction if the corresponding values (mobile shopping pattern) increased. Thus, the use of membership vectors can provide significantly better prediction than raw data. By identifying the data source and analysis methods, our results may contribute to a customized prevention and management service for smartphone dependence based on the membership vectors of the six identified patterns. Accordingly, usage patterns and membership vectors are effective tools for the assessment and prediction of smartphone dependence. Indeed, we can easily capture the usage patterns of smartphone users based on the derived patterns.

This study has some limitations. First, we used a total of 41,683 logs of 48 smartphone users. To generalize the findings, future research should obtain more usage data. Second, although numerous types of smartphone users exist with variations in region and age, our study was limited to users who lived in Seoul and Gyeonggi-do Province and were between the ages of 20 and 39 years. Future research should be conducted using a more diverse population.

Nevertheless, this study has important implications. We used tensor factorization to obtain meaningful patterns from large-scale data. Indeed, various studies in healthcare have attempted to use tensor factorization as a new approach [7,9,20,21]. The analysis of raw data is very challenging, because these data are usually noisy and do not easily map to meaningful concepts (i.e., usage patterns) that are directly used or needed by clinical researchers. Thus, smartphone usage data need to be analyzed using a machine learning method to reduce noise and identify meaningful patterns. Specifically, the user’s usage per day can be expressed as a membership vector with usage patterns after decomposing a tensor. Using the membership vector, we could determine which derived pattern was dominant throughout the day without using log data. Comparison of the raw data and our representation revealed that the membership vector was more compact and interpretable. Using this process, studies such as ours may help establish an intervention service to predict and treat smartphone dependence. Additionally, computational prediction can easily assess smartphone dependence without human effort and provide earlier interventions. Thus, a computational algorithm should be used as a complementary tool with a human decision to improve efficacy. Taken together, our findings provide an intervention guideline for the prediction and treatment of smartphone dependence based on smartphone usage data.

## Conclusions

This study discovered smartphone usage patterns and predicted smartphone dependence according to derived patterns. First, we collected smartphone usage log data. Using tensor factorization, we derived smartphone usage patterns. To predict smartphone dependence, we used the membership vectors as the features of the prediction model. As a result, we identified six usage patterns that significantly predicted smartphone dependence. Moreover, the membership vectors of our usage patterns obtained a significantly better prediction performance than the raw data. These results demonstrate that usage patterns and membership vectors are effective tools for the assessment and prediction of smartphone dependence. Our findings provide an intervention guideline for the prediction and treatment of smartphone dependence based on usage data.

## Supporting information

S1 Appendix S-Scale questionnaire items. (DOCX)

S2 Appendix Details of tensor factorization. (DOCX)
