# Correction: Smartphone dependence classification using tensor factorization

**DOI:** 10.1371/journal.pone.0185506

**Published:** 2017-09-21

**Authors:** The PLOS ONE Staff

There are errors in the Funding section. The publisher apologizes for the errors. The correct funding information is as follows: This research was supported by the Brain Research Program through the National Research Foundation of Korea (NRF) funded by the Ministry of Science, ICT & Future Planning (NRF-2014M3C7A1062893). This work was partly supported by Institute for Information & communications Technology Promotion (IITP) grant funded by the Korea government (MSIP) (No. 2014-0-00147, Basic Software Research in Human-level Lifelong Machine Learning (Machine Learning Center)). The funders had no role in study design, data collection and analysis, decision to publish, or preparation of the manuscript.
