# Supplementary material for: Smartphone dependence classification using tensor factorization
Source: PLoS One. 2017 Jun 21;12(6):e0177629. doi: 10.1371/journal.pone.0177629 (PMC5479529; doi:10.1371/journal.pone.0177629)
Supplement: S1 Appendix — (DOCX) [file pone.0177629.s001.DOCX]

**S1 Appendix. S-Scale questionnaire items.**

| **Factor** | **Question** | **Question number** |  |
| --- | --- | --- | --- |
| Daily life disturbance | Performance degradation in class or office due to smartphone use. | 1 |  |
|  | Using smartphone when I am not supposed to (in classroom, during meeting, etc.). | 5 |  |
|  | People around me tell me that I use the smartphone too much. | 9 |  |
|  | Having difficulties concentrating on job/study due to smartphone use. | 12 |  |
|  | Smartphone use has nothing to do with my performance in class or office. | 15 | reverse |
| Virtual world orientation | Feeling empty when not using my smartphone. | 2 |  |
|  | Feeling more pleasant or excited while using smartphones than while being with friends or family. | 6 |  |
| Withdrawal | Would not be able to stand not having a smartphone. | 8 |  |
|  | Feeling depressed, anxious, or oversensitive when I am not able to use my smartphone. | 14 |  |
|  | Not feeling impatient when not holding my smartphone. | 4 | reverse |
|  | Not being able to work/study without my smartphone. | 11 |  |
| Tolerance | Have tried to shorten smartphone use time but failed all the time. | 7 |  |
|  | Kept using smartphone while thinking about stopping use. | 3 |  |
|  | Using my smartphone longer than I intended. | 13 |  |
|  | Not spending much time using smartphone. | 10 | reverse |
