# Supplementary material for: Smartphone dependence classification using tensor factorization
Source: PLoS One. 2017 Jun 21;12(6):e0177629. doi: 10.1371/journal.pone.0177629 (PMC5479529; doi:10.1371/journal.pone.0177629)
Supplement: S2 Appendix — (DOCX) [file pone.0177629.s002.DOCX]

**S2 Appendix. Details of tensor factorization.**

First, we present preliminary information on tensors and then explain the details of tensor factorization. We use bold uppercase characters for matrices (e.g., $\mathbf{U}$), bold lowercase characters for vectors (e.g., **u**) and bold script font (e.g., $\mathcal{X}$) for a tensor.We represent the $i$-th row of matrix $\mathbf{U}$ as $\mathbf{U}_{i*}$, the $j$-th column as $\mathbf{U}_{*j}$, and the ($i,j$)-th element as $\mathbf{U}_{\mathrm{ij}}$. The two algebraic operations used in this appendix are as follows:

Given $\mathbf{A}\in\mathbb{R}^{I\times J}$ and $\mathbf{B}\in\mathbb{R}^{K\times L}$, the Kronecker product generates a matrix of $\mathrm{size}\left( IK \right)\times(JL)$.

$\mathbf{A}\bigotimes$**B =** $\left[ \begin{matrix} \mathbf{A}_{11}\mathbf{B} & \boldsymbol{\cdots} & \mathbf{A}_{1J}\mathbf{B} \\ \boldsymbol{\vdots} & \boldsymbol{\ddots} & \boldsymbol{\vdots} \\ \mathbf{A}_{I1}\mathbf{B} & \boldsymbol{\cdots} & \mathbf{A}_{IJ}\mathbf{B} \end{matrix} \right]$

Given $\mathbf{A}\in\mathbb{R}^{I\times R}$ and $\mathbf{B}\in\mathbb{R}^{J\times R}$ where **A** and **B** have the same number of columns, the Khatri-Rao product produces a matrix of $\mathrm{size}\left( IJ \right)\times R$.

$\mathbf{A}⨀$**B =** $\left[ \begin{matrix} \mathbf{A}_{*1}\bigotimes\mathbf{B}_{*1} & \boldsymbol{\ldots} & \mathbf{A}_{*R}\bigotimes\mathbf{B}_{*R} \end{matrix} \right]$

A tensor is a multidimensional array. The order of a tensor is the number of dimensions, and each dimension of a tensor is a mode. A *K*-order tensor is denoted by $\mathcal{X}\in\mathbb{R}^{I_{1}\times I_{2}\cdots\times I_{K}}$ where $I_{k}$ is the size of mode *k*. A *K*-order tensor is a rank-one tensor if it can be defined as the outer product “$^{\circ}$” of *K* vectors. A *K*-order tensor can be unfolded into a matrix by reordering the elements of $\mathcal{X}$. This process is called *k*-mode matricization and is denoted by $\mathbf{X}_{(k)}\in\mathbb{R}^{I_{k}\times( \prod_{j\not\equiv k}I_{j} )}$.

Tensor factorization is a widely used dimensionality reduction method that can be used to analyze complex data sets that contain high dimensionality. Tensor factorization aims to approximate the observed tensor using the low-dimensional space. This process is equivalent to minimizing the difference between the observed tensor and the reconstructed tensor from the factor matrices. One of the most common tensor factorizations is CANDECOMP/PARAFAC (CP) decomposition [1, 2]. CP decomposition approximates the observed tensor as a sum of rank-one tensors. Given a *K*-order tensor $\mathcal{X}$ and the number $R$ of latent factors (i.e., usage patterns), the tensor can be expressed as $\mathcal{X}\approx\sum_{r=1}^{R} \lambda_{r}\mathbf{U}_{*r}^{\boldsymbol{(}1\boldsymbol{)}}^{\circ}\mathbf{U}_{*r}^{\boldsymbol{(}2\boldsymbol{)}}^{\circ}\cdots^{\circ}\mathbf{U}_{*r}^{\boldsymbol{(}K\boldsymbol{)}}$ by CP decomposition. Each factor matrix $\mathbf{U}^{(k)}\in\mathbb{R}^{I_{k}\times R}$ represents the latent feature vectors for the *k*-th mode of the observed tensor $\mathcal{X}$. $\lambda\in\mathbb{R}^{R}$ is a vector that indicates the weights of the factor matrices. For an unfolded tensor, $\mathcal{X}\approx\sum_{r=1}^{R} \lambda_{r}\mathbf{U}_{*r}^{\boldsymbol{(}1\boldsymbol{)}}^{\circ}\cdots^{\circ}\mathbf{U}_{*r}^{\boldsymbol{(}K\boldsymbol{)}}$ can be expressed as $\mathbf{X}_{(k)}\approx\mathbf{U}^{\left( k \right)}diag\left( \lambda\right)\mathbf{G}^{T}$, where $\mathbf{G}=\mathbf{U}^{\left( K \right)}⨀\cdots⨀ \mathbf{U}^{\left( k+1 \right)}⨀ \mathbf{U}^{\left( k-1 \right)}⨀\cdots⨀ \mathbf{U}^{\left( 1 \right)}$.

In our study, we used the nonnegative CP alternating Poisson regression (CP-APR) model [3] (the extended CP model), because our observed tensor consisted of count data. Count data can be better represented using a Poisson distribution [3]. Additionally, because CP-APR puts stochastic constraints on the factor matrices, we can easily interpret the elements of the factor matrices. The objective function of CP-APR can be found in [3].

**References in S2 Appendix**

1. Carroll JD, Chang JJ. Analysis of individual differences in multidimensional scaling via an N-way generalization of “Eckart-Young” decomposition. Psychometrika. 1970;35(3):283-319.

2. Harshman RA. Foundations of the PARAFAC procedure: Models and conditions for an “explanatory” multi-modal factor analysis. UCLA Work Papers Phonet; 1970. 1-84.

3. Chi EC, Kolda TG. On tensors, sparsity, and nonnegative factorizations. SIAM Journal on Matrix Analysis and Applications. 2012;33(4):1272-99.
